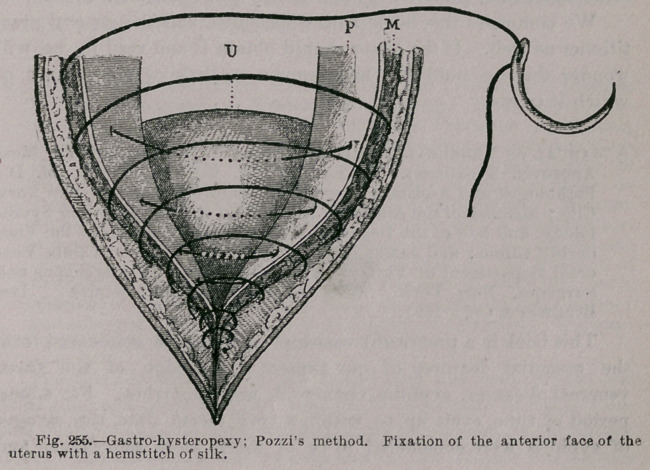# Treatise on Gynecology, Medical and Surgical

**Published:** 1892-05

**Authors:** 


TREATISE ON GYNECOLOGY, MEDICAL AND SURGICAL. By S. POZZI, M. D., Professeur Agrégé de la Faculté de Medecine ; Chirurgien de l'Hôpital Lourcine-Pascal, Paris ; Honorary Fellow of the American Gynecological Society. Translated from the French edition under the supervision of, and with additions by Brooks H. Wells, M. D., Lecturer on Gynecology at the New York Polyclinic ; Fellow of the New York Obstetrical Society, and the New York Academy of Medicine. Volume I. With 305 illustrations and six full-page plates in color. Large 8vo, pp. xxiv.—581. New York: William Wood & Company. 1891.

The literature of gynecology has been enriched within the last ten years by the contributions of many able men, but there is no book that has been issued from the press, either in this country or in Europe, that has impressed us with the degree of perfection, and the conscientious work of the author, that this book of Pozzi must ever be associated with in the minds of his readers. It is based upon Pozzi's large practical experience as a hospital surgeon and teacher, and is, consequently, teeming all through its pages with the elements of originality, which lends a charm to any author's work.

Chapter I., on Antisepsis in Gynecology, brings to our notice, at the very outset, the consideration of a most important question —one of the most important connected with the whole practice of this art. Wherever else antisepsis may be needed, or may not be requisite, certainly here it is of the first importance, and a knowledge of its application is essential to the successful practice of gynecology; hence, this subject very properly forms the opening chapter in the book. How different from the treatises of fifteen or twenty years ago, that began with long and dry chapters on the anatomy of the pelvis, and the organs it contained, which really had little or no place in works on gynecology, because the treatises on anatomy were requisite to supply knowledge in this direction. Following antisepsis comes Anesthesia in Gynecology, which occupies Chapter II., and these two chapters are of the greatest interest. We wish that every physician might read them, whether he be a gynecologist, a surgeon, or a general practitioner.

Pozzi's methods of suture and hemostasis are both interesting and instructive. Though his methods of examination contain nothing beyond the ordinary, his chapter on the Pathology and Etiology of Metritis is excellent. He deals with the question of uterine fibromata in a masterly manner, and his chapters on this subject are full of new thought. In regard to cancer, besides the

Fig. 151.—*A*. Enucleation of an interstitial myoma. *B*. Disposition of sutures after enucleation.

usual operations heretofore recommended in text-books, vaginal hysterectomy has a large place, and Kraske's sacral resection is fully described. The usual space is given to the treatment of dis-

Fig. 255.—Gastro-hysteropexy; Pozzi's method. Fixation of the anterior face of the uterus with a hemstitch of silk.

placements of the uterus, and a great many pessaries and their methods of application are described in detail. The new operations of vaginal hysteropexy and gastro-hysteropexy are fully and amply described and illustrated. Plastic operations of the genital tract are also fully and elaborately dealt with, and the book closes with the subject of Disorders of Menstruation. At the end of each chapter is a voluminous bibliographical reference list, and some of the illustrations are especially to be commended for the clearness with which they bring out the author's meaning. This is especially the case with reference to the methods of suturing, and also the operations upon tumors, the treatment of the pedicle, and all work connected with pelvic and abdominal operations. Some of the old cuts that have done duty with reference to prolapsus of the genital organs might have been omitted without being missed, but, in the main, the book is admirably illustrated, as the two figures which we introduce will indicate. It is printed upon heavy book paper, in large octavo form, containing 581 pages, exclusive of the preliminary matter, and is one of the handsomest books, in its presswork and general make-up, that we have seen for many a year. The translator, Dr. Brooks H. Wells, has contributed not a little to make the book interesting to American readers. The translation is as smooth as though it had been written originally in English, and some of the chapters are marvelously well constructed in their phraseology and tersely put sentences.

We commend this book to the specialist and the general practitioner as well. If the latter should obtain it and read it, he will wonder that he could ever have known so little of the subject of which it treats.

## Figures and Tables

**Fig. 151. f1:**
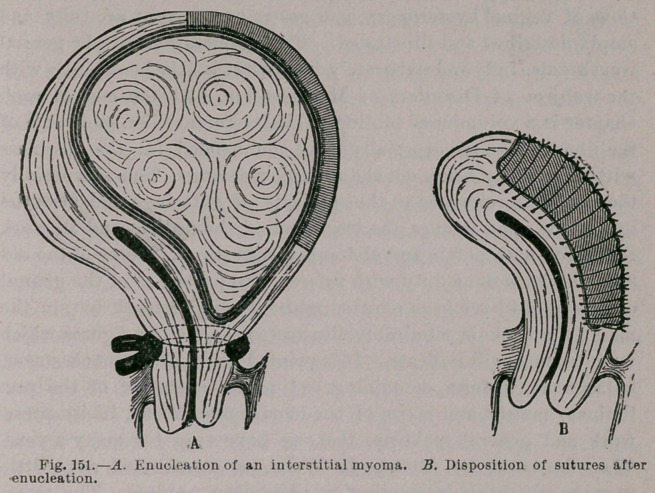


**Fig. 255. f2:**